# 2022: Position of Brazilian Diabetes Society on exercise recommendations for people with type 1 and type 2 diabetes

**DOI:** 10.1186/s13098-022-00945-3

**Published:** 2023-01-02

**Authors:** William Valadares Campos Pereira, Denise Maria Martins Vancea, Ricardo de Andrade Oliveira, Yuri Galeno Pinheiro Chaves de Freitas, Rodrigo Nunes Lamounier, Wellington S. Silva Júnior, Andrea Messias Britto Fioretti, Clayton Luiz Dornelles Macedo, Marcello Casaccia Bertoluci, Roberto Luis Zagury

**Affiliations:** 1 Research Group on Physical Exercise and Non-Transmissible Chronic Diseases from the Physical Education School of the University of Pernambuco (UPE), Recife, Brazil; 2 Physical Education School of the University of Pernambuco (UPE), Avenida Agamenon Magalhães, S/N-Santo Amaro, Recife,, PE CEP 50100-010 Brazil; 3 Department of Obesity and Associated Diseases of the Brazilian Obesity Association (ABESO), Board of Directors of the Rio de Janeiro Society of Exercise Medicine and Sports, Rio de Janeiro, Brazil; 4 Endocrinology Discipline, Federal University of Rio Grande do Norte (UFRN), AV Nilo Peçanha, 620-Petrópolis, Natal, RN CEP 59012-400 Brazil; 5 Faculty of Medicine, Federal University of Minas Gerais, Belo Horizonte, Brazil; 6 Endocrinology Discipline, Department of Medicine I, Faculty of Medicine, Center of Biological Sciences, Federal University of Maranhão (UFMA), Praça Gonçalves Dias, 21, Centro, São Luís, MA CEP 65020-240 Brazil; 7 Center for Exercise and Sports Traumatology, Federal University of São Paulo (UNIFESP), São Paulo, Brazil; 8 Exercise Endocrinology Center of the Federal University of São Paulo (UNIFESP), São Paulo, Brazil; 9 Internal Medicine Department, Federal University of Rio Grande do Sul (UFRGS), Ramiro Barcelos, 2350 Building 12, 4th Floor, Porto Alegre, RS Brazil; 10 Endocrinology Division, Hospital de Clínicas de Porto Alegre (HCPA), Ramiro Barcelos, 2350 Building 12, 4th Floor, Porto Alegre, RS Brazil; 11 Luiz Capriglione State Institute of Diabetes and Endocrinology (IEDE), Rio de Janeiro, Brazil

**Keywords:** Diabetes, Physical exercise, Physical activity, Treatment

## Abstract

**Introduction:**

For individuals diagnosed with diabetes mellitus, the practice of properly oriented physical exercises brings significant benefits to the individual's health and is considered an indispensable tool for metabolic management. The individualization of exercise routines is an essential aspect for therapeutic success, despite the need to consider some general recommendations. This review is an authorized literal translation of the Brazilian Society of Diabetes (SBD) Guidelines 2021–2022, which is based on scientific evidence and provides guidance on physical activities and exercises aimed at individuals with type 1 and 2 diabetes.

**Methods:**

SBD designated 9 specialists from its “Department of Diabetes, Exercise & Sports” to author chapters on physical activities and exercises directed to individuals with type 1 and 2 diabetes. The aim of these chapters was to highlight recommendations in accordance with Evidence Levels, based on what is described in the literature. These chapters were analyzed by the SBD Central Committee, which is also responsible for the SBD 2021–2022 guidelines. Main clinical inquiries were selected to perform a narrated review by using MEDLINE via PubMed. Top available evidence, such as high-quality clinical trials, large observational studies and meta-analyses related to physical activity and exercise advisory, were analyzed. The adopted MeSh terms were [diabetes], [type 1 diabetes], [type 2 diabetes], [physical activity] [physical exercise].

**Results:**

17 recommendations were defined by the members. For this review, it was considered different Evidence Levels, as well as different Classes of Recommendations. As to Evidence Levels, the following levels were contemplated: *Level A*) More than one randomized clinical trial or a randomized clinical trial meta-analysis with low heterogeneity. *Level B*) Meta analysis with observational studies, one randomized clinical trial, sizeable observational studies and sub-groups analysis. *Level C*) Small non-randomized studies, cross-sectional studies, case control studies, guidelines or experts’ opinions. In respect to Recommendation Classes, the following criteria were adopted: *I*. “Recommended”: Meaning there was a consent of more than 90% of the panel; *IIa. “*Must be considered”: meaning there is a general preference of the panel which 70–90% agrees; *IIb. “*Can be considered*”.* 50–70% agrees; *III* Not recommended: There is a consensus that the intervention should not be performed.

**Conclusion:**

Physical exercise aids on the glycemic control of type 2 diabetes individuals while also decreasing cardiovascular risk in individuals with type 1 and 2 diabetes. Individuals diagnosed with diabetes should perform combined aerobic and resistance exercises in order to manage the disease. In addition, exercises focusing on flexibility and balance should be specially addressed on elderly individuals. Diabetes individuals using insulin as therapeutic treatment should properly monitor glycemia levels before, during and after exercise sessions to minimize health incidents, such as hypoglycemia.

## Introduction

Physical activity is defined by any bodily movement derived from using skeletal muscles, resulting on energy expenditure. Conversely, physical exercise can be described as a planned and specific form of a structured physical activity, which can be classified by type, intensity, duration and frequency, with the aim of improving physical conditioning and health [1].

For individuals diagnosed with *diabetes mellitus*, the practice of properly guided physical exercises provides significant benefits for individual’s health, though, it’s considered to be an indispensable tool for metabolic management [2, 3]. Obtaining a regular physical exercise routine is an essential factor in the treatment of type 1 diabetes mellitus (T1DM) and its chronic complications [4].

Even though there are counterindications regarding the direct effect of exercise on glycemic control, in individuals with T1DM, there are significant additional benefits on performing those activities. Benefits may include: reduction of cardiovascular risks, personal welfare promotion, weight control, increase in personal strength and conditioning, reduction on LDL cholesterol levels and triglycerides [5–8]. Thus, physical exercise must be encourage as a fundamental part of diabetes treatment. However, taking in consideration cardiovascular risks, exercise prescription directed for T1DM must be individualized and properly addressed, especially when contemplating higher intensity exercises [9].

T1DM is associated with the following conditions: micro and macrovascular complications, obesity, hypertension, hyperglycemia, dyslipidemia, insulin resistance and sedentarism [10–12]. Regular physical exercise obtains an important role in primary and secondary prevention of cardiovascular diseases, improving general health and well-being [13].

For safety and prevention of adverse events, it is essentially important to assess cardiovascular risk on individuals with T2DM who will begin exercise activities. Even though general recommendation must be considered, individualization of exercise programs is essential for therapeutic success.

This review will analyze what is most relevant in the literature in the context of physical activity and exercise for people with type 1 diabetes mellitus and type 2 diabetes mellitus, with their respective recommendations.

## Methodology

The present revision is an authorized verbatim translation from a session of the 2021–2022 Guidelines of the Brazilian Diabetes Society (SBD). The methodology used has already been published in previous SBD guidelines and was approved by the Central Committee responsible for the SBD Guidelines.

SBD designated 9 specialists from its “Department of Diabetes, Exercise & Sports” to author chapters on physical activities and exercises directed to individuals with type 1 and 2 diabetes. The aim of these chapters was to highlight recommendations in accordance to evidence levels, based on what is described in the literature. These chapters were analyzed by the SBD Central Committee, which is also responsible for the SBD 2021–2022 Guidelines.

Main clinical inquiries were selected to perform a narrative review by using MEDLINE via PubMed. Top available evidence, such as high-quality clinical trials, large observational studies and meta-analyses related to physical activity and exercise advisory, were analyzed. The adopted MeSh terms were [diabetes], [type 1 diabetes], [type 2 diabetes], [physical activity] [physical exercise].

### Evidence levels

Three Evidence Levels were considered:

A. More than one randomized clinical trial or a randomized clinical trial meta-analysis with low heterogeneity.
B. Meta analysis with observational studies, one randomized clinical trial, sizeable observational studies and sub-groups analysis.
C. Small non-randomized studies, cross-sectional studies, case control studies, guidelines or experts’ opinions.

### Recommendation classes

All members from the “Department of Diabetes, Exercise and Sport” and Central Committee received a survey for each Recommendation. Frequency of responses was analyzed, and a Recommendation grade was obtained based on the following criteria:

- Recommended: Meaning there was a consent of more than 90% of the commission.
- Must be considered: There is a general preference of the panel which 70–90% agrees.
- Can be considered: Agreed by the majority. 50–70% agrees.
- Not Recommended: Consensus to not recommend the intervention.

### Physical activity and exercise for type 1 diabetes individuals

For the prescription of exercise in accordance with clinical and physical conditions of T1DM individuals, it is important to assess cardiovascular risk (Table 1) and evaluate exercise modalities by intensity levels (Table 2). Individuals at “high” and “very high” risk should undergo screening to evaluate possible cardiovascular conditions. Table 3 presents a decision flowchart evaluating the need for screening before starting an exercise program in people with diabetes.

**Table 1:**
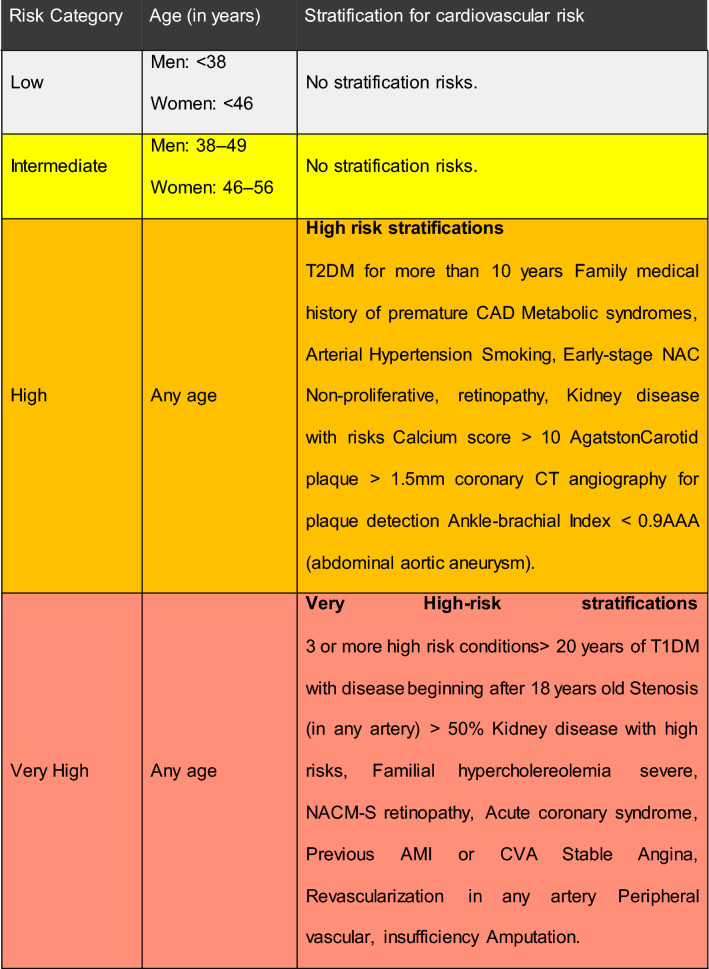
Cardiovascular risk stratification in prescribing physical exercise for diabetic individuals

*T2DM* type 2 Diabetes, *CAD* Coronary arterial disease, *CAN* Cardiovascular autonomic neuropathy, *CT* computer tomographic; *M-S Retinpahty* Medium to severe retinopathy, *T1DM* type 1 Diabetes, *AMI* Acute Myocardial infarction, *CVA* Cerebral vascular accident. The colors in the table can show the level of importance required for decision making

**Table 2:** Suggestion of physical exercise modalities by intensity levels

Intensity	Activity
Low	Easy walk
Moderate	Cycling, slow-paced run, swimming
High	High Intensity Interval Training

**Table 3:** screening of cardiovascular disease in individuals with diabetes without known cardiovascular disease.

Age > 35 years
Type 2 diabetes of > 10 years’ duration
Type 1 diabetes of > 15 years’ duration
Presence of any additional risk factor for coronary artery disease
Presence of microvascular disease (proliferative retinopathy or nephropathy, including microalbuminuria)
Peripheral vascular disease
Autonomic neuropathy

Diabetes care, volume 25, supplement 1, January 2002

Decision making flowchart for screening of cardiovascular disease before exercise in individuals with diabetes. A graded exercise test may be helpful if a patient, about to embark on a moderate-to high-intensity exercise program, is at high risk for underlying cardiovascular disease, based on one of the following criteria:

### Glycemia levels management during physical exercise

Blood glucose levels management and hypoglycemia prevention require special care with adjustments of insulin doses and carbohydrate intake. Such balance will depend on the intensity of exercise and blood glucose levels before and during exercise. Table 3 is a summary of SBD suggestions when referring to pre-exercise blood glucose management. SBD suggestions represent what is considered to be the most appropriate recommendations for hyperglycemia and hypoglycemia prevention. Table 4 suggests continuous glucose monitoring (CGM) methos for the management of glucose levels while performing physical exercise. Tables 5, 6, 7 and 8 suggests insulin bolus dose adjustments for meals that precede exercise by up to 90 min for T1DM individuals.

**Table 4:**
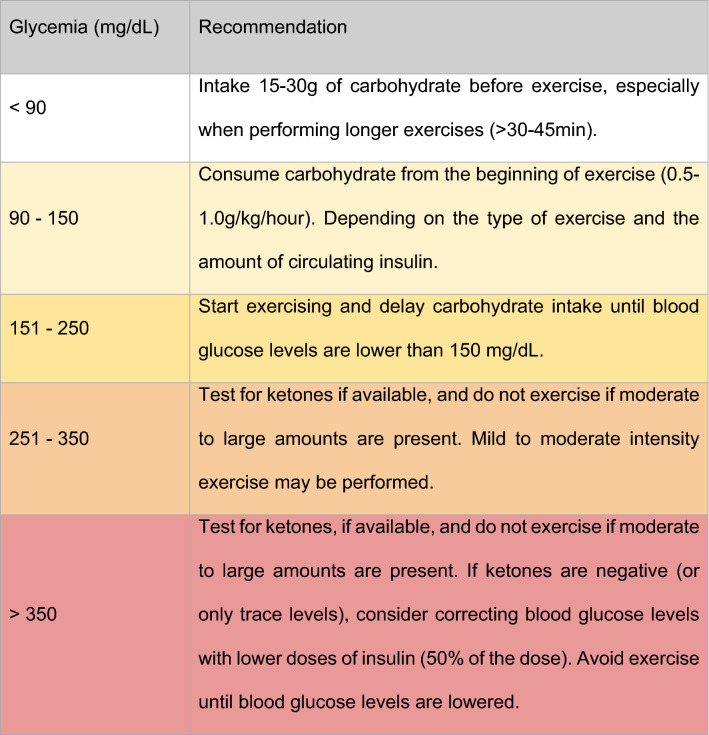
Conduct recommendation based on pre-exercise glucose levels on T1DM

**Table 5:**
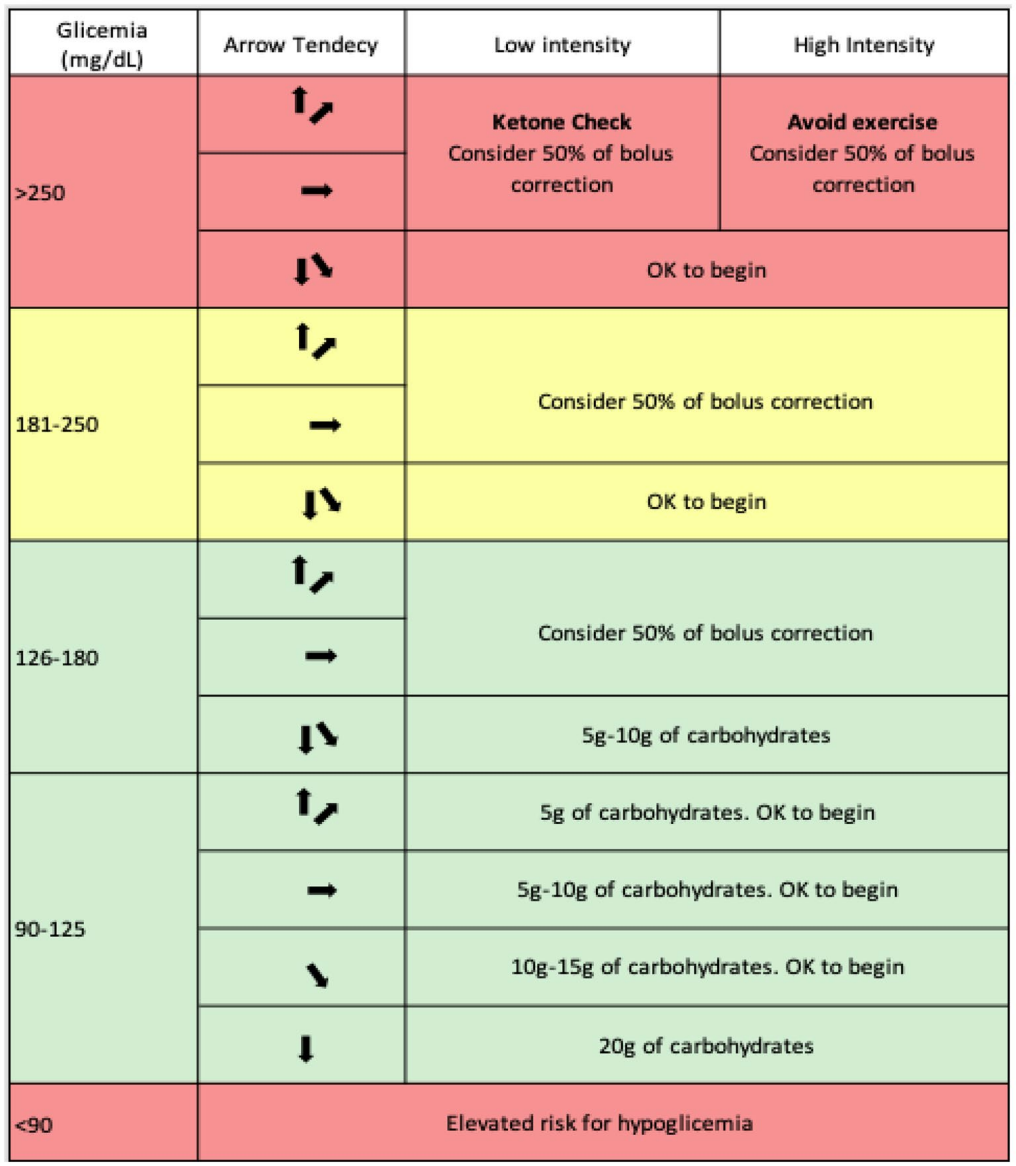
Glucose management during physical exercise adopting CGM

*CGM* Continuous glucose monitoring. Moser [14]. The colors in the table can show the level of importance required for decision making

**Table 6:** Recommendation to decrease insulin bolus on meals that precede exercise by up to 90 min for T1DM

Physical Exercise Intensity	30 min duration	60 min duration
Light Aerobic (~ 25% VO2 max)	− 25%	− 50%
Moderate Aerobic (~ 50% VO2 max)	− 50%	− 75%
Intense Aerobic (70–75% VO2 máx)	− 75%	NA
Intense Aerobic/Anaerobic (> 80% VO2 máx)	No reduction	NA

*NA* Not available

Source: Adapted of Colberg [5] and Yardley [15]

**Table 7:**
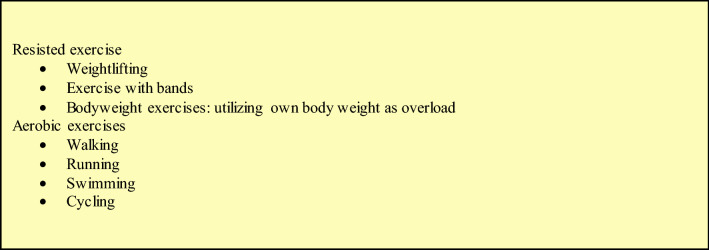
Recommended exercises for T2DM

**Table 8:**
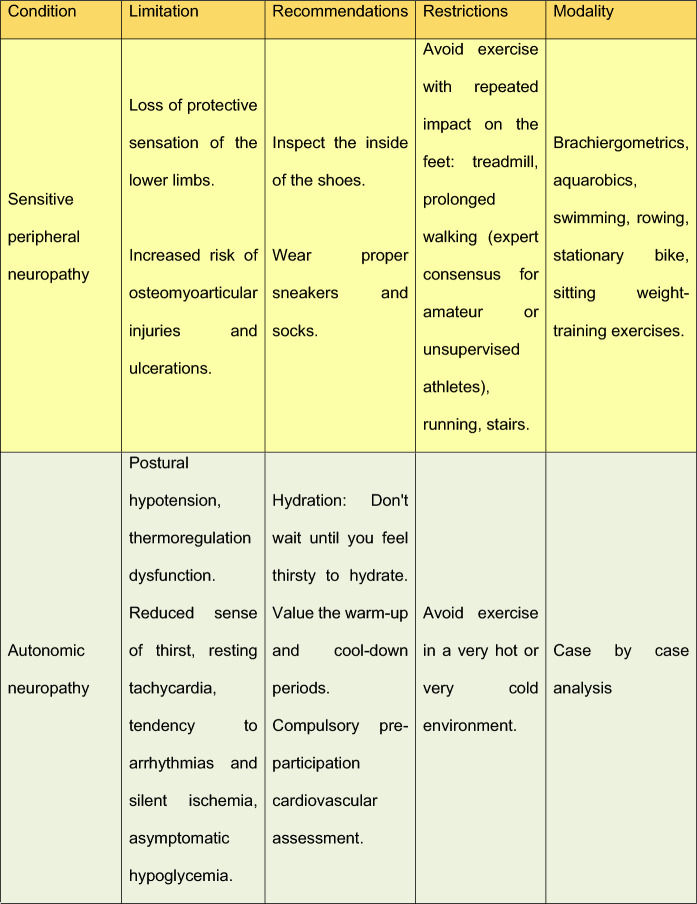

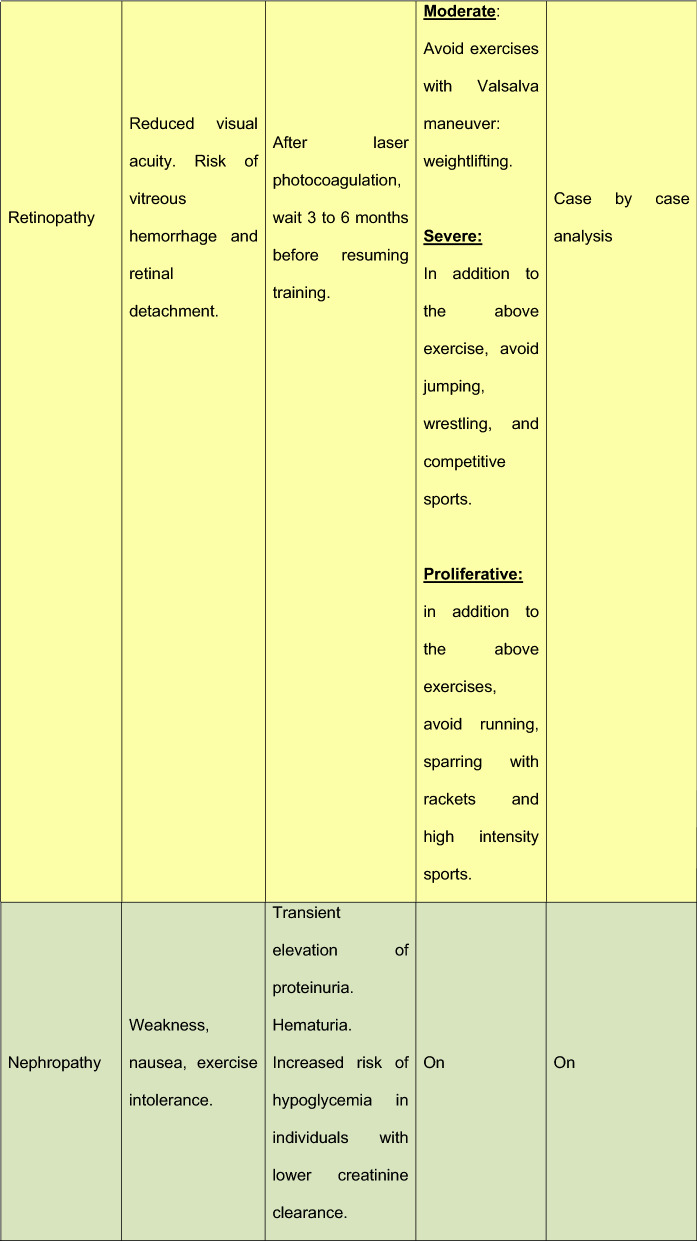
DM microvascular complications and exercise restrictions

**Important note 1:** Changing of background insulin rate or dose overnight after exercise

- After an exercise session, especially if unusual or of long duration, the need for any change in the rate or dose of background insulin overnight after exercise may be considered.

## Recommendations

### Physical exercise indication

#### R1

It is RECOMMENDED that physicians and other health care professionals encourage physical activity and exercise for T1DM individuals as part of their treatment


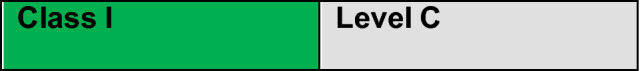


### Summary of evidence

This recommendation is grounded on expert opinion. Health care professionals recognize their role in helping T1DM individuals incorporate physical activity into diabetes management and daily life, however, barriers to successfully integrate these activities are noted. It is important that health care professionals encourage regular participation of adults and children in different physical activity programs [16].

### Cardiovascular risk assessment

#### R2

It is RECOMMENDED that physicians must evaluate cardiovascular risk in all T1DM individuals, in accordance with SBD risk stratification table, before prescribing physical activity or exercise


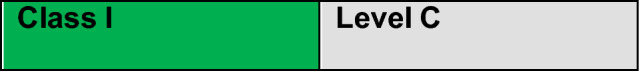


### Summary of evidence

Based on experts’ opinions, screening for cardiovascular diseases in not recommended for low or moderate intensity exercises when referring to asymptomatic T1DM individuals who do not fall into the high or very high-risk categories, since these individuals present a low Predictive Value [1, 17].

High or very high risk T1DM individuals who will begin exercising should be evaluated at least through resting electrocardiogram, and, when indicated, other tests may be performed [17].

**Important note 2:** High intensity exercises

- Prescribing higher intensity exercises should be individualized based on individuals’ physical condition, evaluated by a specialist. Individuals at high or very high risk may have contraindications of specific types of exercise and should be individually evaluated.

**Important note 3**: Exercises and retinopathies

- Individuals with retinopathies should be effectively treated before beginning any exercise programs [17].

**Important note 4:** Peripheral neuropathies and exercise.

- Individuals with peripheral neuropathy should be advised to wear appropriate footwear and to always perform foot self-examination before and after exercise. They may perform resisted physical exercises, such as weightlifting, if there are no ulcers on the feet [17].

#### R3

It is RECOMMENDED that individuals with high or very high risk T1DM, who wish to begin exercise, be initially screened with electrocardiogram in resting position. Depending on individual analysis of each case, additional tests may be requested


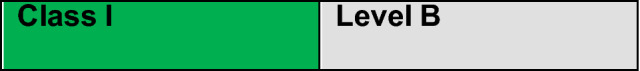


### Evidence summary

A longitudinal cohort study by Soliman et al. [18] aimed to assess the association between the prevalence and incidence of electrocardiographic abnormalities and the development of cardiovascular diseases (CVDs) in T1DM individuals. The study involved 1,306 individuals (mean age 35.5 years ± 6.9 years; 47.7% women). During a mean follow-up of 19 years, 155 participants (11.9%) developed cardiovascular conditions. The study concluded that the presence of major ECG changes is associated with increased risk of CVD in T1DM. This indicates the importance of ECG screening in T1DM individuals in order to properly identify risk for CVD, especially before beginning an intensive exercise program.

#### R4

It is RECOMMENDED that T1DM individuals perform at least 150 min per week of moderate or vigorous intensity aerobic exercise, with no more than two consecutive days resting, for improving fitness and BMI control


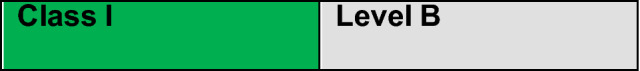


### Summary of evidence

A meta-analysis of 14 randomized controlled studies that evaluated the effect of exercise training in T1DM individuals regarding HbA1c, BMI, and other anthropometric and biochemical parameters, presented no difference in HbA1c levels in adults after exercise. However, BMI was significantly lower [mean difference − 0.39 kg/m2(95% CI − 0.75 to − 0.02; p = 0.03; I2 = 0%)] [6].

The performance of moderate aerobic exercise is associated with lower cardiovascular mortality, and also decrease in all-cause mortality in T1DM and T2DM [18].

An observational study of T1DM adults suggests that higher amounts of physical activity led to lower cardiovascular mortality after an 11.4-year follow up in individuals with and without kidney disease [19].

Children with T1DM should engage in combined aerobic and resistance exercise at least three times per week, for more than 12 weeks, to reduce HbA1C levels [20].

**Important note 5.** Adjustment of insulin doses

- It is necessary for T1DM individuals to adjust insulin doses and carbohydrate intake prior to exercise in order to reduce the risk of hypoglycemia (Tables 3, 4, and 5).

#### R5

In T1DM individuals, aerobic, resistance, or combined training in the same session MUST BE CONSIDERED for improving endothelial function, fitness, and glycemic control


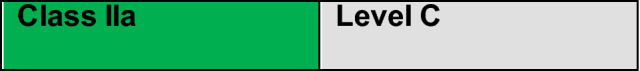


### Summary of evidence

In young and well-conditioned individuals, it should be considered the practice of at least 75 min of weekly high-intensity interval training (HIIT), caring not to remain more than two consecutive days without exercise [21]. HIIT improves peak VO_2_ and arterial stiffness similarly to moderate intensity continuous training, with the advantage of enabling greater glycemic stability and lower hypoglycemia risk when compared to continuous training [22]. In a nourished state, HIIT is a safe, effective, and time-flexible form of exercise for T1DM individuals [15, 21]. It is worth noting that the intensity assessment of aerobic exercise can be evaluated in different ways, by objective or subjective parameters [21].

The study by Boff et al. aimed to compare the effect of high intensity interval training (HIIT) with moderate intensity continuous training (MICT) on endothelial function, oxidative stress and fitness in T1DM individuals. Thirty-six T1DM individuals (mean age 23.5 ± 6 years) were randomized into three groups: HIIT, MICT and a no exercise group (CON). Exercise was performed on a cycle ergometer for 40 min, three times a week for eight weeks, with 50% to 85% of maximum heart rate (HRmax) in HIIT and 50% in MICT. Glycemic control was similar in all groups. In individuals with type 1 diabetes without microvascular complications, after eight weeks HIIT produced significant improvement in endothelial function and fitness compared to MICT with similar glycemic control [23].

Today, with the advance of technology, active video gaming (AVG) can be considered an alternative exercise to aerobic exercise. Gomes et al. [24], compared the effects of AVG and running on cardiovascular and pleasure responses in T1DM individuals. Vessel diameter (VD) and percentage of endothelial function (%EF) were greater in the AVG group, followed by running and resting, 30 min and after 24 h (VD-AVG: 39.6 ± 9.5, 48.8 ± 12.3 and 56.6 ± 13.9 mm; VD-running: 41.5 ± 9.9, 47.4 ± 10.1 and 46.4 ± 12.4 mm; % EF-AVG: 9.6 ± 8.5, 29.6 ± 17.1 and 45.4 ± 25.9%; % EF-run: 7.3 ± 9.4, 14.8 ± 14.1 and 26.8 ± 18.9%; p < 0.05). Enjoyment was also higher in the AVG compared to the running session (9.4 ± 0.7 vs. 7.7 ± 1.6; p < 0.05). AVG showed similar cardiovascular responses when compared to running, however, it also demonstrated improvements in endothelial function and pleasure levels.

In a study performed by Reddy et al. T1DM individuals used a resistance training protocol. In the study, ten adults with T1DM over 12 weeks were allocated to aerobic exercise, resistance exercise, and no exercise groups in an open, crossover study design. The resistance training protocol was 8 to 12 repetitions of five exercises for upper and lower limbs, with strength intensity between 60 to 80% of individuals one maximum repetition. The primary outcome was percentage of time in range (glucose > 3.9 mmol/L and ≤10 mmol/L) for the 24 h after each bout of exercise or rest during the control week. The group undergoing resistance training showed greater time on target 24 h after the intervention (70 vs. 56%; p = 0.013), reinforcing the benefits of resistance training for glycemic control in this population [25].

When compared to aerobic exercise, resistance exercise can increase blood glucose during its execution, determining a lower risk of hypoglycemia, both acutely and post-exercise. Anticipating resistance exercise in relation to aerobic training seems efficient to minimize the risk of hypoglycemia in individuals undergoing insulin therapy [26]. Individualization of the exercise plan is critical for therapeutic success.

The combination of aerobic exercise (brisk walking, running, cycling, swimming) with resistance exercise (free weights, weight-training equipment, elastic bands, or using one's own body weight) and the progressive increase of volume, frequency, load, and intensity have proven effective for the health of individuals with diabetes, promoting a reduction in HbA1c (− 0.1 to − 1.1% in aerobic training, − 0.2 to − 1.6% in resistance training, and 0.1 to − 1.5% in combined training), among other benefits [5, 12, 19].

A systematic review with meta-analysis by Flores et al. [27]. aimed to analyze the effects of physical training on neuromuscular parameters in T1DM individuals. Compared to aerobic training, strength training increased maximal strength (ES: 1.067; p < 0.001), as did combined training (ES: 0.943; p < 0.001).

#### R6

It is RECOMMENDED that T1DM individuals, especially the elderly ones, perform exercises to improve balance and flexibility in order to develop a better range of motion, dynamic and static balance


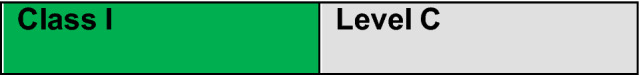


### Summary of evidence

Elderly individuals should prioritize balance and flexibility training. According to individual preferences, exercises such as yoga, tai chi chuan, and joint mobility are recommended for older adults with T1DM [1, 17].

Adults (50 years and older) with diabetes should exercise to maintain and/or improve balance two to three times a week, especially if the patient is diagnosed with peripheral neuropathy. Yoga and tai chi chuan can be included based on individual preferences to increase flexibility and balance [5].

#### R7

In T1DM individuals, glucose monitoring is RECOMMENDED before, during, and after exercise to minimize blood glucose variability and risk of hypoglycemia, consider these suggestions also for people with type 2 diabetes who use insulin or sulfonylureas


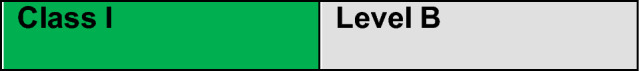


### Summary of evidence

Some precautions can prevent hypoglycemia during exercise and increase the safety of diabetics and those who use insulin, including informing supervisors/counselors and their exercise partners about their clinical condition; Obtaining easy and quick access to fast-absorbing carbohydrates; capillary blood glucose monitoring before, during and after exercise; paying attention to possible symptoms of hypoglycemia [5], consider these suggestions also for people with type 2 diabetes who use insulin or sulfonylureas.

Hypoglycemic events can occur during and after the exercise session. The increased risk of hypoglycemia may be related to improved insulin sensitivity. However, high-intensity exercise can increase blood glucose levels instead of reducing them, and speed sprints can be a strategy to prevent an imminent crisis of hypoglycemia [28]. If pre-exercise blood glucose is elevated or if the effects of counterregulatory hormones replace those of circulating insulin, high-intensity exercise can raise blood glucose [29].

Pre-exercise glycemic values above 250 mg/dL need special management (see Table 4 for suggested management).

#### R8

In T1DM individuals, continuous glucose monitoring (CGM), flash glucose monitoring based on transcutaneous placed glucose oxidase, by transcutaneous placement, CAN BE CONSIDERED during exercise


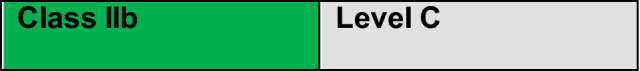


### Summary of evidence

Technology has made it possible to observe more frequently the behavior of interstitial glucose during and after exercise, including at night, and in different sports. Such strategies can decrease the risk and fear of exercise-induced hypoglycemia by providing trends of glycemic variation to help users perform interventions to prevent hypoglycemia and hyperglycemia [30, 31].

Detachment of sensors on the skin, breakage of device filaments, and inability to calibrate may compromise their accuracy and lag-time (lag time in the balance between blood glucose and interstitial glucose values that occurs especially during exercise). The use of these sensors still does not make it possible to dispense with capillary blood glucose monitoring [32].

#### R9

The use of arrow tables on flash monitors for blood glucose management during exercise in T1DM individuals MAY BE CONSIDERED (Table 4)


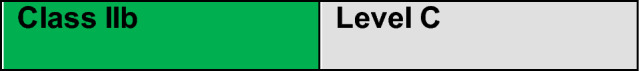


### Summary of evidence

The direction of the glycemic excursions depends, to some extent, on the intensity and duration of the exercise type. Understandably, fear of hypoglycemia is one of the strongest barriers to incorporating exercise into daily life. Risk of hypoglycemia during and after exercise can be reduced when insulin-dose adjustments are made and/or additional carbohydrates are consumed [14].

The use of CGM in exercise studies has allowed the evaluation of trends, by means of arrows and post-exercise (especially nocturnal), for different exercise modalities in T1DM individuals. CGM provides information about late post-exercise responses to help T1DM individuals control their glucose, and is useful as a tool to teach T1DM individuals about exercise responses [14]. Table 4, presents the exercise prescription according to the arrow’s direction.

**Important note 6:** CGMs precision

- Exercise can affect accuracy of the available CGMs, suggesting the need to keep blood glucose in a "cautious" range, above what is generally recommended.
- In addition to assessing blood glucose and considering pre-exercise trend arrows, it is important to assess circulating, active insulin to avoid hypoglycemia during or after exercise.

#### R10

For T1DM individuals using continuous insulin infusion system (CSII), it MAY BE CONSIDERED to reduce the prandial bolus of the meal preceding exercise, reduce the basal infusion rate for a while, or even disconnect it temporarily [33]


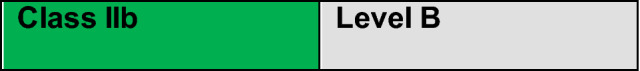


### Summary of evidence

T1DM individuals on continuous subcutaneous insulin infusion (CSII) can reduce the prandial bolus of preceding meal of the exercise, however, the activity must be performed early in the postprandial state (up to 90 min after administration of the prandial bolus). If exercise is not preceded by a meal, individuals may be instructed to disconnect the pump or set a temporary basal rate (50%-80% reduction) at least 90 min before starting exercise [33, 34].

During longer unusual and/or all-day activities (e.g., summer camps or sports clinics), after the activity, individuals may consider a 30% to 50% reduction in basal insulin, throughout the day and evening [35].

**Important note 7**: Insulin dose

- Attention should be paid to avoid excessive reduction of the insulin dose before or during exercise which, associated with carbohydrate intake, can lead to post-exercise hyperglycemia.

### Physical activity and exercise in type 2 diabetes mellitus

The decision to screen asymptomatic T2DM individuals in order to evaluate the presence of CVD before starting an exercise program will depend on the presence of risk stratifiers, presence of cardiovascular symptoms, and intensity of the exercise. Table 3 demonstrated the strategy suggested by the SBD for the indication of screening of cardiovascular disease in individuals with T2DM without known cardiovascular disease.

### Request tests

For individuals eligible for screening before begining exercises, the necessary evaluation tests must be individually determined. The resting electrocardiogram is considered a basic and essential test and should be ordered in all relevant cases. In relation to alternative, more complex, more expensive or more invasive tests, it should be evaluated according to clinical scenario.

### Recommended exercises

For individuals with increased risk of developing T2DM or already in a pre-diabetic state, 150 min of moderate-intense aerobic exercise reduces the risk of developing type 2 diabetes. For individuals with T2DM, combined resistance exercise (at least 1 cycle of 10 to 15 repetitions of 5 or more exercises, two to three sessions per week, on non-consecutive days) and aerobic exercise (at least 150 min per week of moderate or equivalent high intensity, with no more than two consecutive days of no activity) promote significant reductions in HbA1c. Table 1 describes examples of suggested exercises. It is also recommended that adults, especially the elderly, perform physical exercises that improve balance, such as tai chi and yoga, two to three times a week [36, 37].

## Restrictions

For individuals with T2DM and microvascular complications, some restrictions or precautions are necessary. Table 3 describes this necessity.

## Recommendations

### R11

For individuals with increased risk for the developing of T2DM (pre-DM) and to pursue T2DM prevention, physical activity is advocated. It is RECOMMENDED a minimum of 150 min of physical activity in moderate-intensity and a minimum of 7% weight reduction, followed by maintenance of the lost weight is


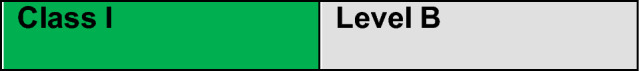


### Summary of evidence

The randomized Diabetes Prevention Program (DPP) study [38] found a greater impact on the incidence of type 2 diabetes as a result of lifestyle intervention, in individuals with impaired glucose tolerance. The study evaluated the performance of physical activity in a minimum of 150 min of moderate-intensity aerobic activity and a minimum of 7% weight reduction with maintenance of weight loss, compared to metformin-based pharmacological therapy. There was a 58% reduction in incidence in the lifestyle change group and a 31% reduction in incidence in the metformin-treated group.

#### R12

Testing for universal screening for cardiovascular disease (CVD) in individuals with T2DM who intend to start exercising is NOT routinely recommended. However, in case there are typical or atypical symptoms of CVD, or in individuals at high or very high cardiovascular risk, it MAY BE RECOMMENDED


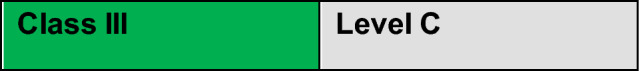


### Summary of evidence

Assessment of cardiovascular risk factors, and a careful physical examination, should be performed with attention to the possibility of atypical presentations of atherosclerotic disease. This is due to screening individuals who will need to undergo other cardiovascular disease tests (Table 3) [1, 39]. Some individuals who plan on training at high intensity or who meet higher risks criteria, may benefit from possible pre-exercise physical stress testing [5, 39].

#### R13

Combined resistance and aerobic exercise is RECOMMENDED for individuals with T2DM: at least one cycle of 10 to 15 repetitions of five or more exercises, two to three sessions per week, on non-consecutive days, and at least 150 min per week of moderate or high-intensity walking, with no more than two consecutive days of no activity (Table 2)


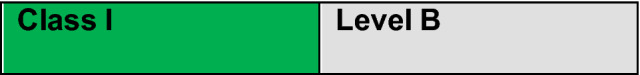


### Summary of evidence

In a systematic review and network meta-analysis, including 2208 individuals with type 2 diabetes, the impact of different physical training modalities on glycemic control, cardiovascular risk factors, and weight loss was evaluated [2]. Both aerobic and resistance exercises promoted significant reduction in HbA1c compared to no exercise (− 0.30% for both interventions) [2]. However, reduction in HbA1c derived by the combined exercise was greater than both modalities (− 0.17% and − 0.23%) compared to aerobic and resistance exercises, respectively [2]. Compared with no exercise, there were benefits of aerobic exercises on blood glucose levels during fasting and lipids, while supervised resistance exercises promoted reduction in systolic blood pressure and total cholesterol. In terms of weight reduction, there were no significant differences between the benefits obtained from aerobic or resistance exercises, neither in combination or not [2].

The optimal strategy for individuals with diabetes should involve combining aerobic exercise with resistance exercise, without remaining more than two consecutive days without activity [5, 39]. After prolonged exercise, glucose uptake remains increased for up to two hours through insulin-independent mechanisms and for up to 48 h through insulin-dependent mechanisms [39, 40, 41]. We suggest that CGM-derived capillary blood glucose or interstitial blood glucose should be considered when planning the use of carbohydrates and insulin during the session that is about to begin [42].

In order to achieve greater weight loss, the amount of exercise can be increased, aiming to reach 300 min to 420 min per week, which also prevents weight gain recovery [43].

#### R14

It is RECOMMENDED that individuals with T2DM or pre-DM reduce the time spent in daily sedentary activities ("sitting time"), in order to reduce cardiovascular risk


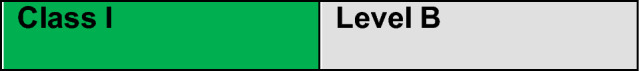


### Summary of evidence

A meta-analysis including a total of nine randomized clinical trials, and 448, 285 participants without diabetes, demonstrated association between time spent sitting ("sitting time") and risk of cardiovascular disease. Statistical significance was held even after adjustment of total structured exercise levels [44].

A meta-analysis of 47 observational cohort studies quantitatively assessed the association between sedentary time in regards of hospitalization, all-cause death, cardiovascular death, diabetes, and cancer in adults regardless of their physical activity. Significant hazard ratio (HR) associations were found with all-cause mortality [(HR, 1.240 [95% CI, 1.090 to 1.410]), cardiovascular disease mortality (HR, 1. 179 [CI, 1.106 to 1. 257]) and onset of T2DM [HR: 1.910 (CI 1.642 to 2.222)]. Prolonged sedentary time was associated with adverse effect outcomes independently of physical activity [45].

A meta-analysis of clinical trials upon individuals with and without diabetes, compared the effect of prolonged sitting time with moments of physical activity throughout the day (INT) to continuous sitting (SIT) on serum glucose, insulin, and triglyceride (TG) levels. At the end, 37 studies were included in the meta-analysis. Regarding glucose, there was a mean standardized difference (SMD) of − 0.54 (95% CI 0.70 to − 0.37, p = 0.00001 in favor of INT compared to SIT; regarding insulin, the SMD was − 0.56 (95% CI − 0.74 to − 0.38 p = 0.0001) and for TG the SMD was − 0.26 (95% CI − 0.44 to − 0.09 p = 0.002). The performance of physical activity during sitting mitigated postprandial insulin, TG and glycemia. Participants with higher BMI demonstrated the greatest attenuation in the referred parameters.

This panel recommends that individuals with diabetes reduce the time spent in daily sedentary activities, interrupting them every 30 min, aiming to achieve activity with an intensity greater than or equal to 1.5 MET [1, 5, 39].

#### R15

It is RECOMMENDED that individuals with T2DM, especially the elderly, practice balance and flexibility training with the goal of reducing falling accidents


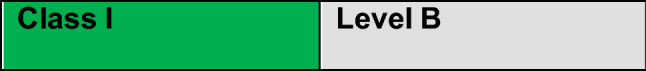


### Summary of evidence

There is evidence that yoga promotes benefits on glycemic control, lipids, and body composition in adults with T2DM [45] and that tai chuan can improve glycemic control, balance, neuropathic symptoms, and some aspects of quality of life in adults with diabetes and neuropathy [46].

There is a lack of robust clinical trials evaluating balance and flexibility training in elderly populations with diabetes. In a systematic review with 159 randomized clinical trials, and data from 79, 193 elderly individuals with or without diabetes [47], multi-component group exercise (including balance and flexibility exercises) was associated with a significant reduction in fall rate (fall rate ratio per person/year [RaR 0.71 (95% CI 0.63 to 0.82)]; [47] 16 trials, 3. 622 participants) and fall risk [RR 0.85 (95% CI 0.76 to 0.96)]; 22 trials; 5,333 participants), as well as home-based multi-component exercise [RaR 0.68 (95% CI 0.58 to 0.80)]; 7 trials, 951 participants and [RR 0.78 (95% CI 0.64 to 0.94)]; 6 trials, 714 participants)] [47].

For tai chi, the reduction in fall rate bordered on statistical significance [RaR 0.72 (95% CI 0.52 to 1.0)]; 5 trials, 1,563 participants), but there was a significant reduction in fall risk [RR 0.71 (95% CI 0.57 to 0.87)]; 6 trials, 1625 participants) [47].

#### R16

It is RECOMMENDED that exercise be accompanied by dietary guidance to maximize its glycemic benefits and, if possible, patients with diabetes should receive written guidance to improve adherence and understanding


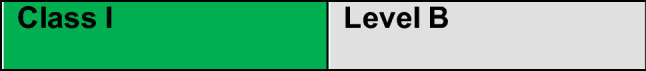


### Summary of evidence

**Fig. 1:**
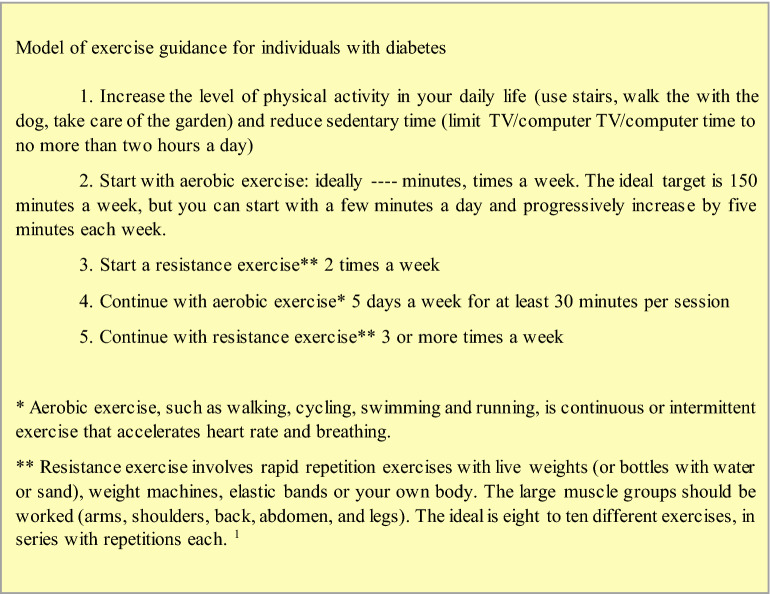
Orientation exercise model for individuals with diabetes. Source: Sports Medicine Outpatient Clinic of the Federal University of São Paulo (UNIFESP)

Exercise should be accompanied by dietary advice to maximize its glycemic benefits and in light of evidence that sedentary individuals who receive formal exercise prescriptions are more likely to exercise than those who only receive verbal advice [48, 49] an exercise guidance model for individuals with diabetes is recommended according to the “exercise guidance model for individuals with diabetes”, Fig. 1.

A small clinical trial (THE GREEN PRESCRIPTION STUDY) including 456 sedentary individuals, randomized two groups to receive verbal counseling or exercise prescription. Recreational physical activity after 6 weeks increased in both groups, but the increase was significantly greater in the group receiving exercise's prescription, n = 218 (p = 0.004).

**Important note 8:**

Determining a daily step goal for the person with diabetes, may be a relevant guidance for initiating exercise. In a systematic review, [50] the use of a pedometer was found to be associated with increased physical activity and reductions in body mass index and blood pressure

#### R17

It is RECOMMENDED that diabetes individuals who practice exercises, and health care professionals, be made aware of the associated risks of indiscriminate use of anabolic steroids and likewise


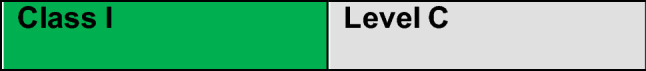


### Summary of evidence

Diabetes individuals who practice exercise must be warned of the associated risks of anabolic steroids and similar drugs abuse, such as: worsening of metabolic control, elevation of LDL-cholesterol, reduction of HDL-cholesterol, polycythemia, left ventricular hypertrophy, cardiomyopathies, arrhythmias, worsening of insulin resistance, hypertension, thrombosis, hepatic peliosis, focal nodular hyperplasia, hepatic adenomas and carcinomas, and various psychiatric disorders. In women, they can also cause hirsutism, acne, alopecia, and clitoromegaly [51–53].
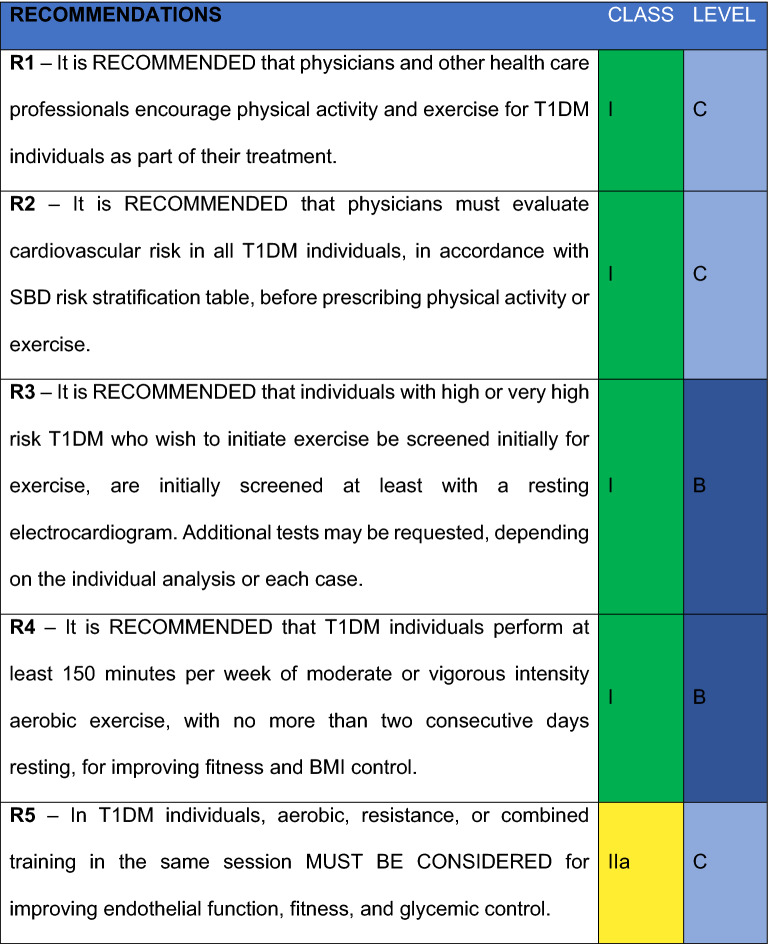

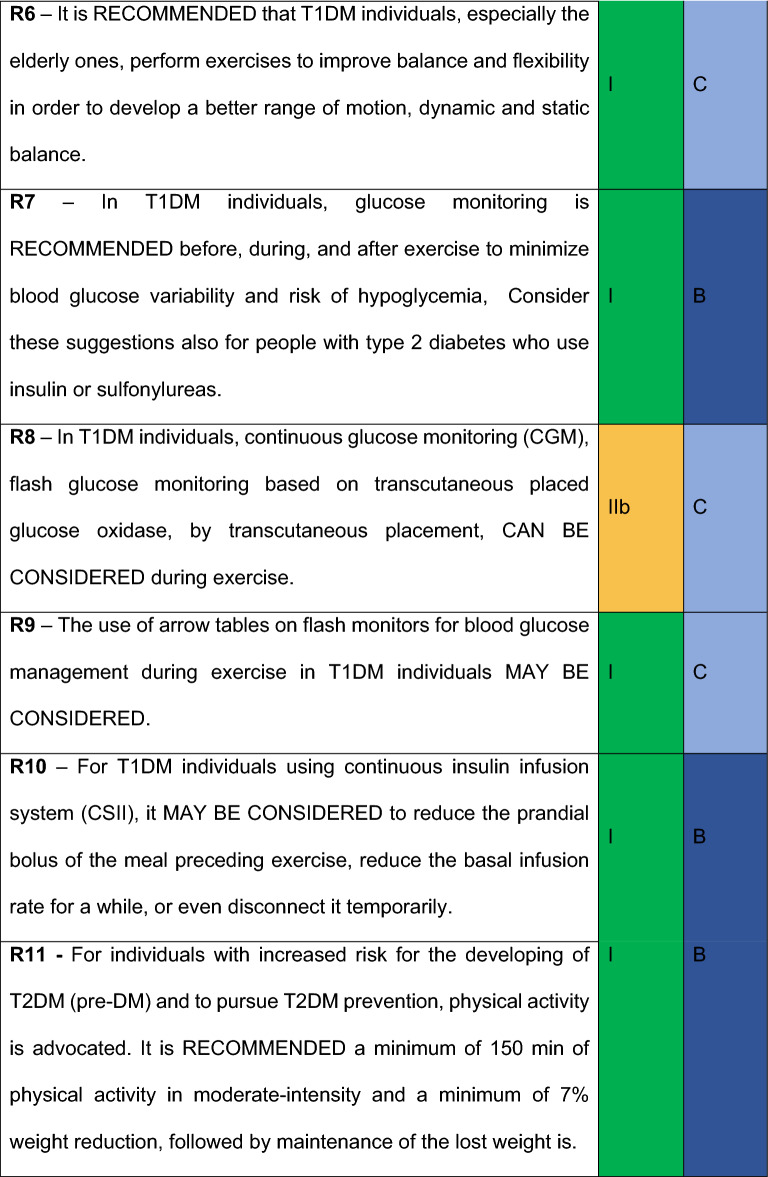

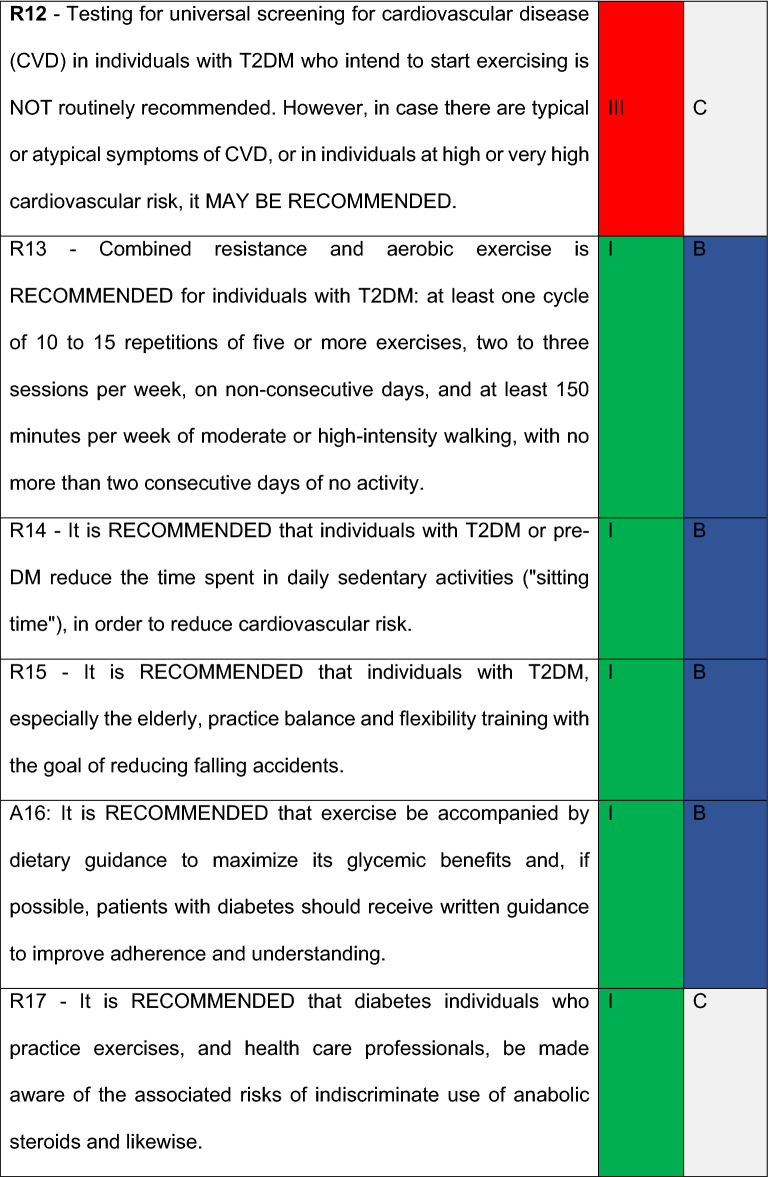


## Conclusion

Exercise helps with glycemic control in individuals with type 2 diabetes and helps reduce cardiovascular risk in individuals with type 1 and 2 diabetes. Individuals with diabetes should perform combined resistance and aerobic exercise. Special attention should be given to balance and flexibility exercises for elderly individuals. Diabetic individuals undertaking insulin should monitor blood glucose levels before, during, and after exercise sessions, in order to minimize complications such as hypoglycemia.

## Data Availability

Data sharing does not apply to this article.

## Abbreviations

LDL: Low Density Lipoprotein
HDL: High Density Lipoprotein
SBD: Brazilian Society of Diabetes
CGM: Continuous glucose monitoring
T1DM: Type 1 diabetes mellitus
T2DM: Type 2 diabetes mellitus
VO_2_ max: Maximum oxygen consumption
ECG: Eletrocardiogram
BMI: Body mass index
HbA1C: Glycated Hemoglobin
HR máx: Maximum heart rate
CSII: Continuous subcutaneous insulin infusion
HIIT: High intensity interval training
